# Longitudinal association of childhood physical activity and physical fitness with physical activity in adolescence: insights from the IDEFICS/I.Family study

**DOI:** 10.1186/s12966-022-01383-0

**Published:** 2022-12-09

**Authors:** Becky Breau, Mirko Brandes, Toomas Veidebaum, Michael Tornaritis, Luis A. Moreno, Dénes Molnár, Lauren Lissner, Gabriele Eiben, Fabio Lauria, Jaakko Kaprio, Stefaan De Henauw, Wolfgang Ahrens, Christoph Buck, on behalf of the IDEFICS/I.Family consortia

**Affiliations:** 1 Department of Human Health and Nutritional Sciences, University of Guelph, Guelph, Canada; 2 Leibniz Institute for Prevention Research and Epidemiology-BIPS, Bremen, Germany; 3 Faculty of Mathematics and Computer Science, University of Bremen, Bremen, Germany; 4 Department of Chronic Diseases, National Institute for Health Development, Tallinn, Estonia; 5 Research and Education Institute of Child health, Strovolos, Cyprus; 6 GENUD (Growth, Exercise, Nutrition and Development) Research Group, University of Zaragoza, Instituto Agroalimentario de Aragón (IA2), Instituto de Investigación Sanitaria de Aragón (IIS Aragón) Zaragoza, Zaragoza, Spain; 7 Centro de Investigación Biomédica en Red de Fisiopatología de la Obesidad y Nutrición (CIBERObn), Instituto de Salud Carlos III, Madrid, Spain; 8 Department of Pediatrics, Medical School, University of Pécs, Pécs, Hungary; 9 School of Public Health and Community Medicine, Institute of Medicine, Sahlgrenska Academy, University of Gothenburg, Gothenburg, Sweden; 10 Department of Public Health, School of Health Sciences, University of Skövde, Skövde, Sweden; 11 Institute of Food Sciences, National Research Council, Avellino, Italy; 12 Institute for Molecular Medicine Finland FIMM, University of Helsinki, Helsinki, Finland; 13 Department of Public Health and Primary Care, Faculty of Medicine and Health Sciences, Ghent University, Ghent, Belgium

**Keywords:** Physical activity, Physical fitness, Accelerometry, PA guidelines

## Abstract

**Background:**

This study aimed to examine associations of early childhood physical fitness and physical activity (PA) with PA during later childhood/early adolescence while accounting for gender differences.

**Methods:**

We selected data of *N* = 4329 children from the IDEFICS/I. Family cohort (age 2.4–11.7 years) with data on baseline fitness and accelerometer measurements. At baseline, physical fitness tests were conducted including Flamingo balance, Backsaver sit and reach, Handgrip strength, Standing Long Jump, 40-m sprint and 20-m Shuttle run (to estimate cardio-respiratory fitness levels). PA was measured with Actigraph accelerometers over 3 days at baseline (ActiTrainer or GT1M) and 7 days at follow-up (GT3X). Evenson cutpoints were used to determine moderate-to-vigorous PA (MVPA) time, and children with ≥60mins/day of average MVPA were deemed as having met WHO guidelines at baseline and follow-up. Linear and logistic regressions were performed to examine longitudinal associations between meeting WHO guidelines, MVPA, and physical fitness tests at baseline with meeting WHO guidelines and MVPA at follow-up. Models were conducted on the entire sample, the sex-stratified sample, and stratified by sex and pubertal status at follow-up.

**Results:**

Results showed that meeting WHO guidelines for MVPA at baseline was positively associated with MVPA (Standardized Beta (B) = 0.13, 95%CI:(5.6;11.1)) and meeting WHO guidelines at follow-up for the entire sample (OR = 2.1, 95%CI:(1.5; 3.14), and stratified by males (OR = 2.5, 95%CI:(1.5; 4.1)) and females (OR = 1.8, 95%CI:(1.0; 3.2)). This was also found for both male pre/early pubertal and pubertal groups but only in the female pre/early pubertal group, and not the female pubertal group (MVPA: B = .00, 95%CI:(− 6.1; 5.6), WHO: OR = 0.61, 95%CI:(0.23;1.6)). Models indicated that Standing Long jump, 40-m sprint, Shuttle run and Flamingo balance at baseline were associated with MVPA and meeting the guidelines at follow-up.

**Conclusions:**

Meeting WHO guidelines and certain fitness tests at baseline were strongly associated with MVPA and meeting WHO guidelines at follow-up, but this association varied with sex and pubertal status. Consequently, these findings underline the importance of ensuring sufficient physical activity in terms of quality and quantity for children at the earliest stages of life.

**Trial registration:**

ISRCTN62310987.

**Supplementary Information:**

The online version contains supplementary material available at 10.1186/s12966-022-01383-0.

## Background

In childhood, particularly during the school aged years, physical activity (PA) is favorably associated with numerous health indicators such as adiposity, cardiometabolic indicators and cognitive performance [1]. Higher intensities of PA such as moderate-to-vigorous PA (MVPA) are more frequently examined and have consistently shown associations with positive health outcomes [1]. Importantly, previous research suggests that PA behaviours can track over time [2, 3]. However, there is a lack of longitudinal studies with long follow-up periods that could provide insight into the timing of the decline of PA or the effectiveness of targeted interventions at various stages of childhood.

To encourage increased levels of PA during childhood, the World Health Organization (WHO) has provided global PA recommendations targeted to children between the ages of 5 and 17 years which recommend a minimum of 60 minutes of MVPA per day [4]. Research supports these recommendations; meeting these guidelines has been shown to be positively associated with lower adiposity, cardiometabolic disease risk and increased physical fitness in children [1].

According to Caspersen et al. [5], physical fitness represents one’s ability to execute physical activities requiring aerobic capacity, endurance, strength or flexibility. Similar to PA, physical fitness has been deemed a positive indicator of health in childhood and adolescence [6]. Although the relationships between PA and physical fitness [1] during childhood and adolescence have been assessed previously, most studies have examined this relationship from a cross-sectional perspective or have solely examined PA as a determinant of fitness. To our knowledge limited longitudinal studies have investigated PA as a predictor of physical fitness [2, 7, 8]; most studies that focused on adolescents [2, 7, 8] used a subjective measure of PA [7] or had a short follow-up period [2]. Clearly, there is a need for further longitudinal investigations of the associations between physical fitness and objectively assessed PA over longer periods of time. Furthermore, as the transition period between childhood and adolescence is marked by developmental changes (e.g. increase in sex hormones, changes in body anthropometry) which may impact PA and physical fitness [9, 10], studies should examine changes in the relationship between PA and fitness based on pubertal status. A previous study found that after a 2-year follow-up, the proportion of children acquiring an average of 60 minutes of MVPA per day decreased by 15% [11]. However, those children, who remained active with at least 60 minutes of MVPA per day, had lower odds of becoming overweight at 2 and 6-year follow-ups. These findings highlight the need for additional longitudinal studies with longer follow-up periods.

Therefore, this study aimed to investigate the longitudinal associations between physical activity and physical fitness from childhood through adolescence. Firstly, we examined whether MVPA and meeting PA guidelines at baseline was associated with the same PA measures at follow-up to examine tracking of PA behaviours over time (Research Question 1). Our second aim was to examine whether baseline performance of physical fitness tests was associated with MVPA and meeting PA guidelines at follow-up (Research Question 2). To examine how this association differed between males and females before and after puberty, all associations were conducted for i) the entire sample, ii) stratified by sex, and iii) stratified by sex and pubertal status at follow-up.

## Methods

### Study design and population

The IDEFICS/I.Family cohort, retrospectively registered under ISRCTN62310987, is a population-based study which aimed to examine lifestyle-related diseases in children and adolescents from eight European countries including Belgium, Cyprus, Estonia, Germany, Hungary, Italy, Spain, and Sweden [12]. The IDEFICS study (Identification and prevention of dietary- and lifestyle-induced health effects in children and infants) comprised a baseline survey (T0, September 2007 – June 2008, *n* = 16,299 children aged 2.2–9.9 years) and two follow-up surveys at T1 (September 2010 – May 2011, *n* = 11,041 children aged 4–11.9 years), including 2555 newly recruited children [13]; and at T2, only conducted to assess dissemination of the intervention messages (by mail), not the full survey protocol. The follow-up surveys revealed only weak effects of the intervention [14], thus data from T0 and T1 were pooled together to incorporate a larger sample. Participants from T0 and T1 of the IDEFICS study as well as their parents and siblings were invited to participate in an enhanced third follow-up, the I. Family study (T3, in 2013–2014), where *n* = 7105 participated in the third follow-up which aimed to gather additional information pertaining to the entire family [15]. Requirements with respect to ethics approval and written or verbal (for children aged under 12 years) consent was obtained from local ethics committees by participating centres in all eight countries.

**Fig. 1:**
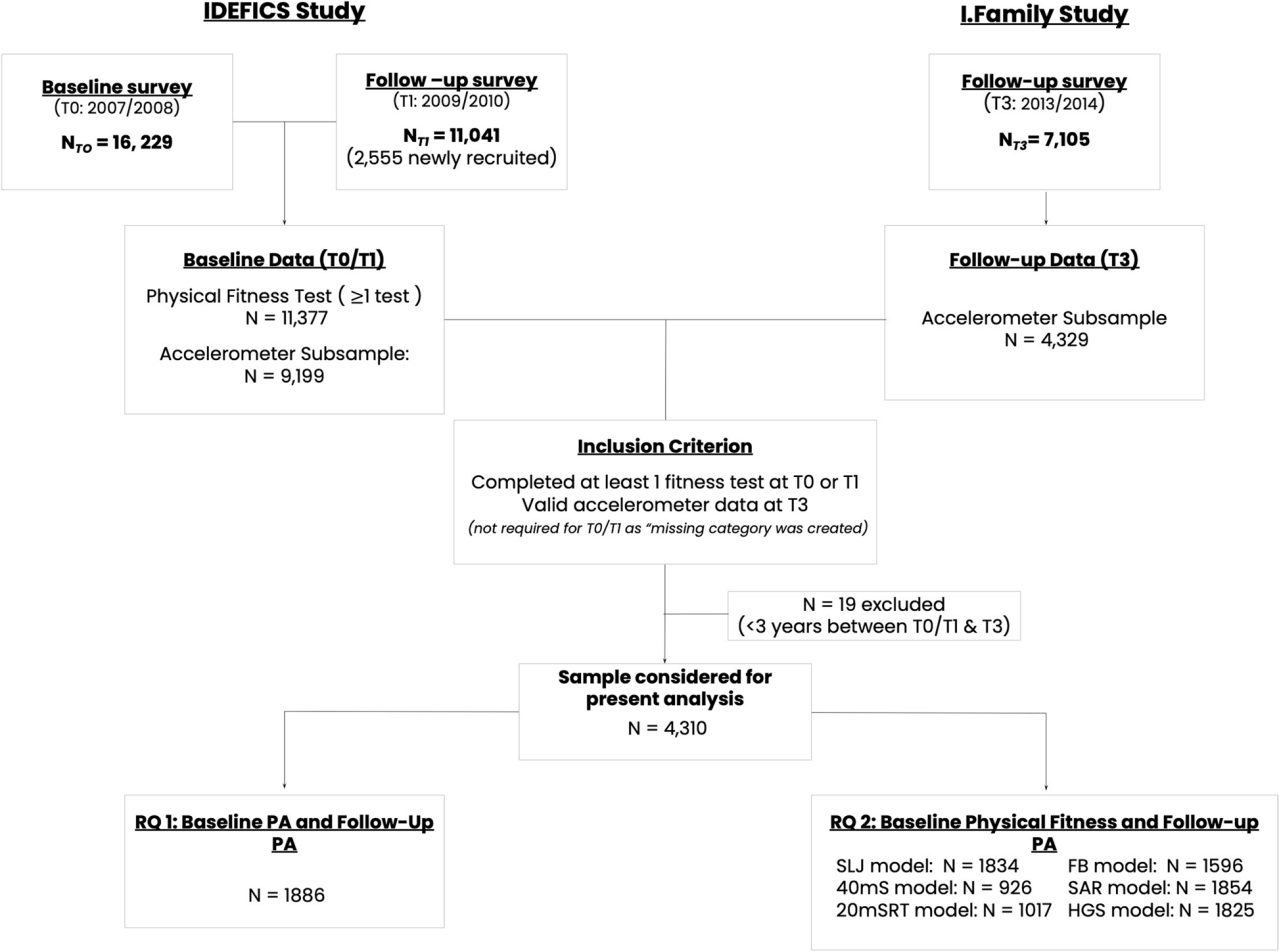
Flow chart of the IDEFICS /I. Family sample population used in this analysis

The present analysis included longitudinal data from the IDEFICS baseline and follow-up surveys (T0/T1) as well as data from the I.Family survey (T3). Accelerometer data was collected in a subsample at T0/T1 and T3 and physical fitness data was only collected at T0/T1. As these measures were only collected in subsamples of the IDEFICS and I. Family populations, a total of *n* = 4, 329 children were considered for this analysis. From this smaller sample, an additional *n* = 19 children were excluded as there was less than 3 years between the baseline (T0 or T1) and follow-up (T3) assessments, as such a total of *n* = 4310 children were included in the present descriptive analysis. Given our research questions about the associations between physical activity and physical fitness from childhood through adolescence, children must have i) completed at least one of the fitness tests at T0/T1, ii) provided parent survey information at T0/T1 and T3 and iii) had valid accelerometer data at T3 to be included in the final analyses. Children were not required to have accelerometer data at T0/T1 as a “missing category” was created for those who were not included in the accelerometry subsample. A total of *n* = 1, 280 children provided valid accelerometer data at T0/T1 and *n* = 1, 894 children provided valid accelerometer data at T3. The number of children included in the sample for the present analysis who completed fitness tests ranged from 2106 (40 m Shuttle run Test) to 4230 (Backsaver sit and reach test). Figure 1 provides a flow-chart of the sample size based on the IDEFICS / I.Family cohort and considered exclusion criteria.

### Covariate information

In all centres, children were asked to wear light clothing and remove shoes while height and weight measurements were taken by nurses who were trained to follow standardized protocols. Height was measured to the nearest 0.1 cm using a clinical Seca 225 stadiometer (Seca, Hamburg, Germany). Weight was measured to the nearest 0.1 kg using a BC420 SMA scale (Tanita, Amsterdam, Netherlands). Body mass index (BMI) was classified (thin/normal or overweight/obese) according to cut-offs established by Cole and Lobstein [16].

Survey data was collected from parents regarding age, sex, income, and socio-economic status (SES). The highest educational level of parents was classified according to the international Standard Classification of Education (ISCED) to assess SES [17]. ISCED classifications were collapsed into low (ISECD 0–4) and high (ISCED 5+) categories. An additional missing category was created for any participants who did not have data reported on ISCED status. During I. Family assessments, information on pubertal development (i.e., voice change in boys and occurrence of the first menstrual period in girls) was used to classify children into pre/early pubertal or pubertal status. In some countries, Tanner stages were also assessed, and where information on pubertal status was missing, Tanner stages 1&2 were classified as pre-pubertal and Tanner stage 3 was classified as pubertal [18].

### Physical activity

Physical activity was assessed using Actigraph accelerometers (Actigraph, LLC, Pensacola, FL, USA) worn on the right hip during waking hours only. Full details regarding accelerometer data processing from the IDEIFICS study were previously reported by Konstabel et al. [19]. During the IDEFICS (T0/T1) protocol, children were asked to wear either the ActiTrainer or GT1M device for at least 3 days, including 1 weekend day. For the I. Family (T3) protocol, children were asked to wear either the GT1M or GT3X+ device for a period of 7 days. Previous research has confirmed that activity counts from the vertical axis are comparable between the GT1M and GT3X+ devices [20], thus activity counts from only the vertical axis were used for this analysis. Devices were set to collect data at a sample rate of 30 Hz and data was downloaded in 15 sec epochs using ActiLife software (version 6, ActiGraph, Pensacola, FL). It should be noted that some centres inadvertently used 60 sec epochs for a considerable portion of their initial data processing, therefore all data were reintegrated into 60 sec epochs. Non-wear time was identified using a 60 minute window, to detect 30 minutes of consecutive zero counts, with a 2 minute tolerance for breaks of non-zero counts as defined by Choi et al. [21]. Participants were included if they had a minimum of 360 minutes of valid wear time on at least one weekday and one weekend day to compromise between accuracy and sample size as discussed in Konstabel et al. [19]. Remaining valid data were then scored using the Evenson cut points scaled up for 60 sec epochs (SED: 0–100; LPA: 101–2295; MPA: 2296–4011; and VPA: 4012+) [22]. For the present analysis, average minutes per day spent in TPA (LPA + MPA + VPA), MVPA and average valid wear time (mean minutes per day) were calculated. Children were then classified as having met the WHO recommendations for physical activity for ages 5–17 years if they obtained at least 60 mins of MVPA per day based on their average minutes spent in MVPA [4]. As no children under 5 years were included in the PA analyses, only the WHO guidelines for 5–17-year-olds were used. A missing category was created for children who did not have valid accelerometer data at T0/T1; only children with accelerometer data at T3 were included in the analyses involving physical activity and fitness.

### Physical fitness tests

Physical fitness testing was completed during T0 /T1 and the five test items were largely based on the ALPHA health-related fitness test battery which has shown reliability in children and adolescents [23–25]. Test items included: the Flamingo Balance test (FB), Backsaver Sit and Reach test (SAR), Handgrip Strength test (HGS), Standing Long Jump test (SLJ) and 40-m Sprint test (40mS). Additionally, the 20-m Shuttle Run test (20mSRT). To be included in the present analysis, children must have completed at least one of these fitness tests. All testing protocols have been described previously [23, 26] and full details are provided in Supplementary Table S1.

Briefly, during the FB test, children stood on one foot and the number of times their free leg touched the ground within 1 minute was recorded. For the SAR test, children reached as far as possible with one leg out straight, the furthest distanced reached (cm) was recorded. During the HGS test, children squeezed a dynameter (TKK 5101; Takei, Tokyo, Japan) as hard as possible and results were recorded in kilogram force (kgf) and then converted into Newtons (N). For the SLJ test, children were instructed to jump as far as possible and land with feet together, the distance was from the starting point to the most posterior heel was recorded (cm). For the 40mS test, children ran a 40-m distance as fast as possible, speed was recorded in kilometers per hour (km/h). Finally, the 20mSRT test was administered to assess cardio-respiratory fitness, children were instructed to run 20-m, back-and-forth while matching their pace to beep signals. Children continued until reaching fatigue or failing to complete the distance before the beep on two occasions. Estimate values of VO2 Max calculated using the Leger equation [27] were used for analysis which has shown to be a valid and reliable measure of VO2 max in children [27, 28]. Note that participants from Italy and Hungary did not undergo this test, and the survey center in Hungary used a different protocol that could not be unified with results from other centres.

### Statistical analyses

Descriptive statistics in terms of means, standard deviations and proportions were calculated to describe the study participant characteristics (T0/T1 and T3), fitness test performance (T0/T1) and PA variables (T0/T1 and T3).

### Research question 1: is baseline PA associated with follow-up PA?

To assess the longitudinal associations of PA behaviours, we first conducted linear regressions to assess the longitudinal association of meeting PA guidelines at T0/T1 with MVPA at follow-up. Subsequently, logistic regressions were conducted to predict whether children who met WHO guidelines at baseline were more likely to meet WHO guidelines at follow-up. These regressions were adjusted for age, sex, country, ISCED, income, BMI category all at T0/T1; and time between T0/T1 and T3 (gap), pubertal status and valid wear time at T3. These models were conducted for the entire sample, stratified by sex and thirdly, stratified by sex and pubertal status at T3.

### Research question 2: is baseline physical fitness associated with follow-up PA?

To assess the associations between baseline levels of physical fitness with PA measures at follow-up, performance on each fitness test at T0/T1 were all individually regressed onto average time spent in MVPA at T3 using linear regressions. Subsequently, logistic regression models were conducted to predict whether children with higher performance on physical fitness tests at T0/T1 predicted higher likelihood of meeting WHO guidelines at T3. These models were adjusted for age, sex, country, ISCED, income, BMI category, meeting WHO guidelines all at T0/T1; and time between T0/T1 and T3 (gap), pubertal status and valid wear time at T3. All regressions were conducted for the entire sample, stratified by sex and thirdly, stratified by sex and pubertal status at T3.

Level of significance for all statistical analyses was set to α = 0.05 to obtain 95% confidence limits (95%CL) as a precision measure of beta estimates. In addition, standardized Beta coefficients were calculated. As a sensitivity analysis, we compared baseline characteristics of children who provided PA data based on accelerometry and who were included in this study with children who participated in T3 but had no PA measurements taken, due to different reasons. All statistical analyses were conducted in IBM SPSS Statistics (Version 26.0) analytics software.

## Results

Descriptive characteristics for age, anthropometry, PA, and fitness test performance for T0/T1 and T3 can be found in Table 1. The average age at T0/T1 was 7.5 years and was 12.6 years at follow-up. Children acquired an average of 53 mins of MVPA per day at T0/T1 and an average of 47 mins of MVPA per day at T3. In general, more males met the PA guidelines of ≥60 mins of MVPA at both T0/T1 (47% of males, 25% of females) and T3 (30% of males and 17% of females). Performance on most fitness tests was similar between sexes, with females reaching slightly further on the Backsaver Sit and Reach (21.3 cm vs 18.9 cm) and males jumping further on the Standing Long jump test (111 cm vs 102 cm). For both males and females, average MVPA was higher at T0/T1 than at T3, (Females: 48 mins (T0/T1) vs 43 mins (T3), Males: 59 mins (T0/T1) vs 51 mins (T3)).

**Table 1:** Descriptive variables from IDEFICS and I. Family, for the whole sample and stratified by sex

	All (*n = 4310*)				Male (*n = 2149*)				Female (*n = 2161*)			
	n	Mean	SD	Range	N	Mean	SD	Range	N	Mean	SD	Range
**IDEFICS T0/T1 (baseline)**												
Age [years]	4310	7.5	1.0	2.40–11.7	2149	7.5	1.0	3.3–11.6	2161	7.6	1.0	2.40–11.7
BMI z-score	4310	0.51	1.2	−5.3–4.6	2149	0.51	1.2	−5.3–4.6	2161	0.51	1.1	−4.7–4.0
BMI Category (Cole)												
Underweight/Normal	3237				1620				1617			
Overweight/Obese	1073				529				544			
ISCED Category												
*Low*	2091				1031				1060			
*High*	2040				1027				1013			
*Missing*	179				91				88			
Valid Wear Time [min./day]	1273	762	143	434–1335	604	768	149	435–1335	669	757	136	441–1257
MVPA [min./ day]	1273	53	24	1–145	604	59	25	3–145	669	48	21	1–125
WHO PA guidelines met?^a^												
*Yes*	448				283				165			
*No*	825				321				504			
*Missing*	3037				1545				1492			
FB [touchdowns]	3338	7.8	6.9	5–52	1563	9.0	7.5	2–52	1775	6.8	6.1	2–39
SAR [cm]	4230	20.1	5.5	0–41	2101	18.9	5.4	0–35	2129	21.3	5.3	0–41
HGS [N]	4166	107.9	29.0	24.5–261.3	2071	113.5	29.9	24.5–261.3	2095	102.2	26.9	24.5–238.8
SLJ [cm]	4208	106	23	10–197	2089	111	24	10–197	2119	102	22	24–188
40mS [km/h]	2106	9.4	1.2	6.6–15.5	1029	9.2	1.2	6.6–14.0	1077	9.5	1.1	6.8–15.5
20mSRT [VO2 Max]	2387	47.7	2.8	37–60	1159	48.2	3.0	38–60	1228	47.1	2.5	37–56
**i.Family T3 (follow-up)**												
Age [years]	4310	12.6	1.3	7.7–16.2	2149	12.6	1.3	9.2–15.8	2161	12.7	1.3	7.7–16.2
Years between T0/T1 and T3	4310	5.1	1.0	3.0–8.8	2149	5.1	1.0	3.0–8.8	2161	5.1	1.0	3.0–7.0
Pubertal Status												
Pre/Early Pubertal	1751				870				881			
Pubertal	2260				1110				1150			
Missing	299				169				130			
Valid Wear Time [min./day]	1886	780	101	436–1350	916	777	106	453–1350	970	783	96	436–1262
MVPA Time [min./day]	1886	47	20	0–135	916	51	212	0–135	970	43	18	6–125
WHO PA guidelines met?^a^												
*Yes*	442				279				163			
*No*	1444				638				807			
*Missing*	2424				1232				1191			

^a^WHO PA guidelines for 5–17-year-olds of 60 minutes of MVPA. Classified using average MVPA values

### Research question 1: is baseline PA associated with follow-up PA?

Results from the linear regression models using WHO guidelines at baseline to predict MVPA and WHO guidelines at follow-up are presented in Table 2**.** Meeting WHO guidelines for PA at T0/T1 was a significant predictor of higher MVPA at T3 (Standardized Beta (B) = 0.13, 95%CI:(5.4; 11.1)). When stratified by sex, meeting WHO guidelines at T0/T1 predicted higher MVPA at T3 for both males (B = 0.16, 95%CI:(5.3; 14.1)) and females (B = 0.12, 95%CI:(3.5; 11.3)). In the stratified samples by sex and pubertal status at T3, meeting the WHO guidelines at T0/T1 was a strong predictor of higher MVPA at T3 in the pre/early pubertal groups which was more pronounced in females (B = 0.24, 95%CI:(9.1; 20.1)) compared to the male group (B = 0.17, 95%CI:(3.4; 18.2)). Meeting WHO guidelines at T0/T1 was a strong predictor of higher MVPA at T3 in the male pubertal group (B = 0.12, 95%CI:(1.1, 12.8*)*) but *not* in the female pubertal group.

**Table 2:** Linear/logistic regressions between WHO guidelines at baseline with MVPA and meeting WHO guidelines at follow-up

**Does meeting WHO guidelines at baseline predict MVPA at follow-up?**						
	**n**	**ß**	**B**	**95% CI**	**Adj R**^**2**^	***P***
Whole Sample ^a^	1886	8.3	.13	5.4, 11.1	.24	**< 0.001**
*Stratified by Sex*						
Males ^b^	916	9.7	.16	5.3, 14.1	.20	**< 0.001**
Females ^b^	970	7.4	.12	3.5, 11.3	.24	**< 0.001**
*Stratified by Sex and Pubertal status*						
Pre/Early Pubertal Males ^c^	399	10.8	.17	3.4, 18.2	.21	**0.004**
Pubertal Males ^c^	473	6.9	.12	1.1, 12.8	.16	**0.020**
Pre/Early Pubertal Females ^c^	419	14.6	.24	9.1, 20.1	.29	< 0.001
Pubertal Females ^c^	507	−.26	.00	−6.1, 5.6	.21	0.931
**Does meeting WHO guidelines at baseline predict meeting WHO guidelines at follow-up?**						
	**n**	**OR**		**95% CI**	**R**^**2**^	***P***
Whole Sample ^a^	1886	2.2		1.5, 3.1	.21	**< 0.001**
*Stratified by Sex*						
Males ^b^	916	2.6		1.5, 4.3	.19	**< 0.001**
Females ^b^	970	1.9		1.1, 3.4	.20	**0.027**
*Stratified by Sex and Pubertal status*						
Pre/Early Pubertal Males ^c^	399	3.8		1.6, 8.9	.24	**0.02**
Pubertal Males ^c^	473	1.7		.87, 3.5	.17	0.118
Pre/Early Pubertal Females ^c^	419	5.0		2.2, 11.7	.27	**< 0.001**
Pubertal Females ^c^	507	.61		.23, 1.6	.20	0.327

*ß* Beta coefficient, *B* Standardized Beta coefficient, *95% CI* 95% Confidence Interval, *OR* Odds Ratio

“No” is references category for Meeting WHO guidelines

^a^ Models adjusted for Age, Sex, Country, ISCED, Income, BMI category, Gap, Pubertal Status, Valid Wear Time at T3

^b^ Models adjusted for Age, Country, ISCED, Income, BMI category, Gap, Pubertal Status, Valid Wear Time at T3

^c^ Models adjusted for Age, Country, ISCED, Income, BMI category, Gap, Valid Wear Time at T3

Results from the logistic regressions indicated that children who met the WHO guidelines at T0/T1 were more likely to meet WHO guidelines at T3 (OR = 2.1, 95%CI:(1.5; 3.1)). For males and females, meeting WHO guidelines at T0/T1 showed a significantly higher chance of meeting WHO guidelines at T3 (males: OR = 2.6, 95%CI:(1.5; 4.3)*,* females: OR = 1.9, 95%CI:(1.1; 3.4)). When stratified by sex and pubertal status at T3, children who met the WHO guidelines at T0/T1 were more likely to meet guidelines at T3 in males (OR = 3.8, 95%CI:(1.6; 8.9)) and females (OR = 5.0, 95%CI:(2.2; 11.7)) pre/early pubertal groups. However, this was not found in both pubertal groups, as meeting WHO guidelines at T0/T1 was not associated with meeting WHO guidelines at T3.

### Research question 2: is baseline physical fitness associated with follow-up PA?

Results from the linear regression models using physical fitness test performance at baseline to predict MVPA at follow-up are presented in Tables 3 and 4. As shown in Table 3, analysis on the whole sample revealed that better performance on the SLJ (B = 0.07, 95%CI:(0.02; 0.11)), 40mS (B = − 0.09, 95%CI:(− 2.8; − 0.38)), and 20mSRT (B = 0.10, 95%CI:(0.27; 1.17)), at T0/T1 predicted higher MVPA at T3. When stratified by sex, better performance in SLJ, 40mS and 20mSRT predicted higher MVPA at T3 in the male group, while alower performance in the FB test predicted higher MVPA at T3 for the female group (Table 3). In the stratified samples by sex and pubertal status, results for the pubertal male group showed that higher performance on the 40mS test was a strong predictor of higher MVPA at T3 (B = − 0.18, 95%CI:(− 5.5; −0.91)) and better SLJ performance was associated with higher MVPA at T3. In the female pubertal group, lower performance on the FB test was the only strong predictor of increased MVPA at T3 (B = 0.13, 95%CI:(0.13; 0.68)).

**Table 3:** Linear regressions of associations between physical fitness at baseline and MVPA at follow-up

Predictor	**Whole Sample**						**Males** ^b^						**Females** ^b^					
	n	ß	B	95% CI	Adj R^2^	*P*	n	ß	B	95% CI	Adj R^2^	*P*	n	ß	B	95% CI	Adj R^2^	*P*
FB ^a^	1597	.03	.01	−.12, .18	.24	.688	731	−.17	−.05	−.39, .05	.21	.130	866	.25	.08	.05, .45	.24	**.014**
SAR ^a^	1854	.08	.02	−.08, .24	.24	.339	895	.07	.02	−.18, .32	.20	.576	959	.01	.00	−.19, .21	.24	.914
HGS ^a^	1825	−.12	−.02	−.50, .03	.24	.537	882	.00	.00	−.06, .05	.20	.958	943	−.03	−.04	−.08, .02	.24	.254
SLJ ^a^	1834	.06	.07	.02, .11	.25	**.005**	886	.10	.11	.03, .17	.21	**.004**	948	.02	.03	−.03, .08	.24	.398
40mS ^a^	926	−1.6	−.09	−2.8, −.38	.21	**.010**	438	−2.4	−.13	−4.2, −.63	.17	**.008**	488	−.57	−.03	−2.2, 1.1	.19	.506
20mSRT ^a^	1081	.72	.10	.27, 1.17	.16	**.002**	471	1.0	.14	.38, 1.7	.16	**.002**	546	.10	.01	−.57, .77	.17	.760
	*Stratified by Sex and Pubertal status*																	
	**Males**																	
Predictor	**Pre/Early Pubertal**									**Pubertal**								
	n			ß	B	95% CI	Adj R^2^		*P*	n	ß		B		95% CI	Adj R^2^	*P*	
FB ^c^	296			−.17	−.06	−.48, .15	.23		.304	409	−.23		−.07		−.54, .09	.17	.164	
SAR ^c^	385			−.13	−.03	−.53, .27	.22		.528	468	.20		.05		−.13, .53	.16	.232	
HGS ^c^	384			−.08	−.10	−.16, .01	.22		.083	456	.05		.08		−.03, .13	.16	.175	
SLJ ^c^	384			.09	.09	−.02, .19	.21		.124	459	.09		.11		.00, .18	.17	.048	
40mS ^c^	164			−2.0	−.11	−5.2, 1.2	.15		.222	252	−3.2		−.18		−5.5, −.91	.13	**.006**	
20mSRT ^c^	197			1.1	.14	−.05, 2.2	.12		.062	246	.76		.10		−.16, 1.7	.13	.106	
	**Females**																	
Predictor	**Pre/Early Pubertal**									**Pubertal**								
	n			ß	B	95% CI	Adj R^2^		*P*	n	ß		B		95% CI	Adj R^2^	*P*	
FB ^c^	360			.04	.01	−.27, .36	.29		.785	471	.40		.13		.13, .68	.20	**.004**	
SAR ^c^	412			.20	.05	−.13, .52	.29		.230	504	−.06		−.02		−.33, .22	.21	.689	
HGS ^c^	406			.02	.03	−.07, .11	.29		.602	495	−.06		−.09		−.12, .01	.21	.084	
SLJ ^c^	408			.09	.10	−.01, .18	.30		.076	500	.00		.00		−.07, .08	.21	.939	
40mS ^c^	190			−1.2	−.08	−4.1, 1.6	.18		.387	275	.06		.00		−2.3, 2.4	.18	.961	
20mSRT ^c^	237			.50	.06	−.54, 1.5	.22		.341	279	−.04		−.01		−.96, .88	.16	.927	

*ß* Beta coefficient, *B* Standardized Beta coefficient, *95% CI* 95% Confidence Interval, *FB* Flamingo Balance, *SAR* Backsaver Sit & Reach, *HGS* Handgrip Strength, *SLJ* Standing Long Jump, *40mS* 40-m Sprint, *20mSRT* 20-m Shuttle Run

^a^ Models adjusted for Age, Sex, Country, ISCED, Income, BMI category, WHO guidelines met at baseline, Gap, Pubertal Status, Valid Wear Time at T3

^b^ Models adjusted for Age, Country, ISCED, Income, BMI category, WHO guidelines met at baseline, Gap, Pubertal Status, Valid Wear Time at T3

^c^ Models adjusted for Age, Country, ISCED, Income, BMI category, WHO guidelines met at baseline, Gap, Valid Wear Time at T3

**Table 4:** Logistic regressions of associations between physical fitness at baseline and meeting WHO guidelines at follow-up

Predictor	**Whole Sample**					**Males** ^b^					**Females** ^b^				
	n	OR	95% CI	Adj R^2^	*P*	n	OR	95% CI	Adj R^2^	*P*	n	OR	95% CI	Adj R^2^	*P*
FB ^a^	1597	1.0	.98, 1.0	.20	.881	731	.98	.95, 1.0	.19	.138	866	1.0	1.0, 1.1	.20	.065
SAR ^a^	1854	1.0	.99, 1.0	.21	.157	895	1.0	.98, 1.0	.19	.515	959	1.0	.98, 1.1	.21	.358
HGS ^a^	1825	.98	.93, 1.0	.20	.442	882	.98	.92, 1.0	.18	.591	943	.97	.88, 1.1	.206	.470
SLJ ^a^	1834	1.0	1.0, 1.0	.21	**.009**	886	1.0	1.0, 1.0	.20	**.004**	948	1.0	.99, 1.0	.21	.570
40mS ^a^	926	.90	.77, 1.0	.22	.185	438	.82	.67, 1.0	.24	.063	488	.98	.75, 1.3	.21	.890
20mSRT ^a^	1017	1.0	.99, 1.1	.18	.072	471	1.1	1.0, 1.2	.18	**.012**	546	.98	.89, 1.1	.18	.685
		*Stratified by Sex and Pubertal status*													
		**Males**													
Predictor		**Pre/Early Pubertal**						**Pubertal**							
		n	OR	95% CI	Adj R^2^		*P*	n	OR		95% CI		Adj R^2^	*P*	
FB ^c^		296	.99	.95, 1.0	.25		.566	409	.96		.92, 1.0		.18	.082	
SAR ^c^		385	1.0	.96, 1.1	.23		.668	468	1.0		.97, 1.1		.17	.567	
HGS ^c^		384	.92	.83, 1.0	.24		.118	456	1.0		.92, 1.1		.16	.663	
SLJ ^c^		384	1.0	1.0, 1.0	.24		.112	459	1.0		1.0, 1.0		.19	**.017**	
40mS ^c^		164	.93	.67, 1.3	.30		.681	252	.75		.56, 1.0		.21	.053	
20mSRT ^c^		197	1.1	.99, 1.2	.25		.075	246	1.1		.97, 1.2		.15	.183	
		**Females**													
Predictor		**Pre/Early Pubertal**						**Pubertal**							
		n	OR	95% CI	Adj R^2^		*P*	n	OR		95% CI		Adj R^2^	*P*	
FB ^c^		360	.99	.93, 1.0	.28		.743	471	1.1		1.0, 1.1		.20	**.011**	
SAR ^c^		412	1.0	.99, 1.1	.28		.084	504	1.0		.95, 1.1		.20	.820	
HGS ^c^		406	1.0	.90, 1.2	.28		.576	495	.92		.81, 1.0		.20	.225	
SLJ ^c^		408	1.0	.99, 1.0	.29		.281	500	1.0		.99, 1.0		.21	.874	
40mS ^c^		190	.96	.63, 1.5	.29		.860	275	1.2		.78, 1.7		.25	.454	
20mSRT ^c^		237	1.0	.88, 1.2	.23		.752	279	.96		.82, 1.1		.25	.556	

*95% CI* 95% Confidence Interval, *OR* Odds Ratio, *FB* Flamingo Balance, *SAR* Backsaver Sit & Reach, *HGS* Handgrip Strength, *SLJ* Standing Long Jump, *40mS* 40-m Sprint, *20mSRT* 20-m Shuttle Run

^a^ Models adjusted for Age, Sex, Country, ISCED, Income, BMI category, WHO guidelines met at baseline, Gap, Pubertal Status, Valid Wear Time at T3

^b^ Models adjusted for Age, Country, ISCED, Income, BMI category, WHO guidelines met at baseline, Gap, Pubertal Status, Valid Wear Time at T3

^c^ Models adjusted for Age, Country, ISCED, Income, BMI category, WHO guidelines met at baseline, Gap, Valid Wear Time at T3

As shown in Table 4 results from logistic regressions showed that some physical fitness tests had statistically significant associations with meeting WHO guidelines at T3, but OR were ≤ 1. Specifically, results indicated that SLJ performance was positively associated with meeting WHO guidelines at T3. Males with better performance on the SLJ and 20mSRT at baseline had higher chances for meeting WHO guidelines at T3, but no associations were found in the female group. In the male pubertal group, performance on the SLJ was significantly positively associated with meeting guidelines at T3 (OR = 1.0, 95CI:(1.0; 1.0)). Similarly, pubertal females with lower performance on the FB test had a significantly higher chance of meeting WHO guidelines in T3 (OR = 1.1, 95%CI:(1.0; 1.1)).

Sensitivity analysis revealed no substantial differences in descriptive statistics of baseline characteristics and covariates comparing children who provided PA measurements at follow-up and children who did not (results not shown).

## Discussion

The current study aimed to examine the longitudinal associations between childhood physical fitness and physical activity during later childhood/early adolescence and how this association differed between males and females. Results indicated that baseline PA was a predictor of follow-up PA, and certain physical fitness tests were associated with follow-up PA.

### Baseline physical activity and follow-up PA

Results from this study support previous research that indicates PA behaviours can track over time [2, 3, 29]. Recently, Potter et al. [29] found that PA time in children aged 4–5 years moderately tracked over a period of 3 years while Jaakkola et al. [2] found that accelerometer-derived MVPA from 11-year-old children at baseline was significantly associated with MVPA 1 year later. While these results are interesting, Potter et al. [29] used a questionnaire to assess PA and did not collect information on PA intensity and Jaakkola et al. [2] used a short follow-up period of just 1 year. Our results showed that meeting PA guidelines during T0/T1 was strongly associated with average time spent in MVPA and children who obtained greater than or equal to 60 minutes of MVPA at T0/T1 were more likely to do so an average of 5.1 (1.0) years later.

These associations were found for both sexes, however, results differed when the sample was stratified by sex and pubertal status at T3. Meeting PA guidelines at T0/T1 remained a strong predictor of MVPA at T3 in males regardless of pubertal status, but it did not predict MVPA at T3 in females within the pubertal group. Additionally, males and females in the pre/early pubertal groups who met PA guidelines at T0/T1 were more likely to meet guidelines at T3, but this was not found for males and females in the pubertal groups. One possible explanation for this finding is that PA does not track over time but rather it declines during adolescence. Research supports this idea as numerous studies have shown that PA declines from childhood to adolescence [9, 30, 31] and that PA declines more rapidly in girls than boys [9, 32, 33]. Consequently, results from the most recent Health Behaviour in School-aged Children (HBSC) survey in Europe and Canada suggest that PA levels are consistently lower in girls compared to boys at all ages, and that the decline in PA during adolescence is steeper in boys compared to girls [34]. However, PA estimates were derived from self-reported questionnaires for this survey. Findings from Farooq et al. [35] suggest that PA does not start declining during adolescence, but rather propose that PA is *already* declining by the time children reach school-age. Therefore, another possible explanation of our findings is that PA levels were the highest at T0/T1 in the pre/early pubertal groups and remained high enough to meet guidelines for children who stayed in the pre/early pubertal groups. For children who transitioned through puberty between T0/T1 and T3, or who had already reached puberty at T0/T1, their levels of PA had been declining for years meaning they may have had enough PA to marginally meet PA guidelines at T0/T1 but due to continued declines in PA, they did not acquire enough MVPA to meet guidelines at T3. Previous studies did not account for pubertal status in their analyses [30, 35], therefore the role of puberty in the change of PA from childhood to adolescence should be further investigated. Furthermore, pubertal status should be considered during design intervention and additional consideration should be given to strategies or developmental theories concerning PA in pubertal females. Whether PA tracks over time or PA begins to decline at a young age, the findings from our study have important implications for future interventions and emphasize the importance of targeting PA habits during early childhood.

### Baseline physical fitness and follow-up PA

Results from our statistical analyses involving fitness tests performed T0/T1 showed that better performance on the SLJ, 40mS and the 20mSRT were associated with increased MVPA at T3 and increased SLJ performance was statistically significantly associated with meeting PA guidelines at T3. Although the OR for this association was equal to 1, the information gained from the regression analyses indicates that SLJ, as an indicator of lower extremities power, is associated with increased MVPA at follow-up and therefore SLJ performance may be an important contributor for meeting PA guidelines at baseline. The results are concurrent with findings from Jaakkola et al. [7] who found that physical fitness in a sample for 12-year-olds was longitudinally associated with PA 6 years later, however, they used a composite score for fitness and PA was assessed via a questionnaire in their analyses.

Our results from the sex stratified sample showed that associations between fitness at T0/T1 and MVPA or meeting guidelines at T3 differed between males and females. In the male group, increased SLJ, 40mS and 20mSRT (cardio-respiratory fitness) performance were associated with increased MVPA at T3. Associations between SLJ and 40mS, and meeting WHO guidelines at T3 were statistically significant, however, the odds of meeting guidelines were not increased for those with higher test performance. For all female participants and pubertal females, only their performance on the FB test was associated with MVPA at T3, however the direction of this association indicated that an increased number of touchdowns (decreased balance) was associated with increased MVPA. More research is needed to clarify this association and additional measures of balance (static and dynamic) should be examined. Furthermore, it remains unclear why performance on other fitness tests (SLJ, 40mS and 20mSRT) were associated with follow-up PA in males and not females. Future research is needed to elucidate the mechanisms behind the observed sex differences in associations between specific fitness tests and PA.

Previous research has found sex differences in the associations between fitness and PA; Huotari et al. [8] showed that increased fitness levels during adolescence predicted higher PA engagement during adulthood in males and not females. Additionally, Jaakkola et al. [2] found that cardiorespiratory fitness as measured by the 20mSRT, was longitudinally associated with MVPA in boys and not girls, although participants were, on average, older at baseline (11.36 years compared to our average of 7.5 years). In the sex and pubertal status stratified groups, fitness tests were only statistically significantly associated with meeting guidelines in the pubertal groups. This could potentially be explained by the increased strength of the relationship between fitness and physical activity during middle to late childhood as postulated by Stodden et al. [10]. However, the variation in growth and maturation during adolescence makes it difficult to fully comprehend the association between fitness measures and PA [36].

### Strengths and limitations

This study has some major strengths such as a relatively large sample size, a longitudinal study design with a long follow-up period and the use of accelerometers to obtain objective measures of physical activity. However, some limitations need to be discussed. The first limitation is that the data is selected form a large non-randomized cohort underlying selection bias, due to loss-to follow-up and the option for the participants to opt out single measurements as described in [15]. Overall, bias was addressed by including confounders to adjust the regression analyses. In addition, physical fitness data was not collected at T3 in the I.Family study, preventing us from examining the bidirectionality of the relationship between physical activity and physical fitness over time. An additional limitation is that although our study design incorporated a large timeframe between T0/T1 and T3, more follow-ups with shorter time intervals may have provided more insight into the effect of puberty on the relationship between PA and fitness with follow-up PA. Furthermore, we did not include measures of pubertal status at T0/T1, and therefore future studies should analyze the effect of the transition through puberty on the association between fitness and PA. Further, children of the IDEFICS study did not randomly attend fitness tests, but depending on the given schedule and provided consent. Unfortunately, accelerometer measurements at T3 could not fully cover children who also provided accelerometer measurements at baseline resulting in a smaller sample size. However, comparing children who did and who did not provide PA measurements at T3, no substantial differences were revealed. Overall, loss to follow-up in the IDEFICS / I.Family cohort was found to be associated with overweight or obesity at baseline, but overall attrition did not seem to affect the distribution of BMI at follow-up [37].

Future research should examine the types of physical activities regularly engaged in by male and female children and how sports participation varies with sex and development (e.g., pubertal status).

## Conclusions

This study found that children meeting WHO guidelines for PA of greater than or equal to 60 minutes of MVPA per day at baseline had higher MVPA and higher chance of meeting WHO guidelines at follow up. In other words, more active children at a younger age were more likely to be more active at an older age, which is a highly relevant finding for the early development of an active lifestyle. Additionally, some fitness tests such as Standing Long Jump, Flamingo balance test, 40-m sprint and 20-m Shuttle Run test of cardiorespiratory fitness were longitudinally associated with MVPA, and meeting PA guidelines and these associations varied with sex and pubertal status. Results from the present study emphasize the critical importance of engaging children in PA during early childhood to increase the likelihood of sustained PA engagement later in life and that habitual physical activity should be supported by structured physical activities (e.g., during daycare facilities, schools, physical education, sport clubs) to strengthen children’s fitness and health perspectives.

## Supplementary Information

**Additional file 1: Supplementary Table S1**. Detailed description of Physical Fitness Testing Protocols 27, 28, 38.

## Data Availability

The datasets generated and analyzed during the current study are not publicly available because this study is based on highly sensitive data collected in young children. But interested researchers can contact the IDEF ICS and I. Family consortia (http://www.ideficsstudy.eu/Idefics/ and http://www.ifamilystudy.eu/) to discuss possibilities for data access.

## Abbreviations

PA: Physical Activity
MVPA: Moderate to Vigorous Physical Activity.
WHO: World Health Organization
SLJ: Standing Long Jump
LJ: Lateral Jumping
OLS: One Legged Stand
SAR: Backsaver Sit and Reach
FB: Flamingo Balance test
HGS: Hand Grip Strength
40mS: 40-m Sprint
20mSRT: 20-m Shuttle Run Test
OR: Odds Ratio
