# Supplementary material for: Longitudinal association of childhood physical activity and physical fitness with physical activity in adolescence: insights from the IDEFICS/I.Family study
Source: Int J Behav Nutr Phys Act. 2022 Dec 9;19:147. doi: 10.1186/s12966-022-01383-0 (PMC9733271; doi:10.1186/s12966-022-01383-0)
Supplement: Supplementary file 1 — Additional file 1: Supplementary Table S1. Detailed description of Physical Fitness Testing Protocols 27, 28, 38. [file 12966_2022_1383_MOESM1_ESM.docx]

## Supplementary Material

Supplementary Table S1: Detailed description of Physical Fitness Testing Protocols

| **Physical Fitness Test** | **Ability assessed** | **Testing Protocol** |
| --- | --- | --- |
| **Flamingo Balance (FB)** | single leg balance | - Children stood on one foot, bent free leg backwards and held their foot. - Children were instructed to maintain position for 1 minute - Each child was given one practice trial. - The number of attempts needed to stand on one leg for one full minute was recorded - Children were excluded if they touched down >15 times within the first 30 seconds. - Score = sum of attempts on both legs; lower scores indicate better performance. |
| **Backsaver Sit & Reach**  **(SAR)** | hamstring flexibility | - Testing required a standard box with a scale on top, each leg was assessed separately. - Children sat with their testing leg straight, foot flat against the box, while the other leg was relaxed in a bent position and slowly reach as far forward as possible. - Children had 2 attempts per leg, the furthest distance reached was recorded for each leg. - Score = avg max distance reached of both legs; higher scores indicate better performance. |
| **Handgrip Strength**  **(HS)** | maximal upper body isometric force production | - Children stood with both feet shoulder width apart and extend arms by their side. - Holding a dynamometer (TKK 5101; Takei, Tokyo, Japan), children were told to squeeze as hard as possible, without letting the dynamometer touch any part of their body. - Dynamometers were adjusted for sex and hand size for each child (38). - The scale started at 5 kg, to children who did not reach this were given a score of 2.5 kg - The test was performed twice per hand, and the highest score per hand was recorded. H - Score = avg of right and left handgrip strength; higher scores indicate better performance. |
| **Standing Long Jump**  **(SLJ)** | lower limb explosive strength | - Children jumped as far as possible without falling and land with both feet together. - Attempts were invalid if the child touched the ground with their hands upon landing. - Distance was measured from the point of take-off to the back of the most posterior heel. - Two attempts were given - Score = furthest distance jumped (cm). Higher scores indicate better performance. |
| **40-meter Sprint**  **(40mS)** | maximum running speed | - 40 meters was marked off by 5 cones placed 10-meters apart. A running lane was created by placing another row of 5 cones, 3 meters apart from the first row of marker cones. - Children were instructed to run as fast as possible upon a starting signal. The test was performed twice, - Score = fastest time; lower scores indicate better performance. |
| **20-meter Shuttle-Run (20mSRT)** | cardio-respiratory fitness  (VO2 Max) | - Children were instructed to run back-and-forth between two lines that were 20 meters apart while matching their pace to a recording of beep signals. - The child continued to run until reaching fatigue or failing to reach the line before the beep on two consecutive occasions. - Protocols were unified using the Leger protocol to estimate VO2 max, which has shown good test-retest reliability in children (coefficient = 0.89) and adults (coefficient = 0.95) (27). This test has also shown good validity against portable gas analyzers (28)   Unification of Protocols:   - 4 different versions of this test were applied during field assessments. The *Multistage fitness test* (sports coach UK) was applied in Germany, Estonia and Cyprus; the *Leger test* (CAEP Quebec Faca) was applied in Spain and Hungary, the *Multistage fitness test* was applied in Sweden and the *Uithouding Shuttle run test* was conducted in Belgium. - The Leger protocol (27) was used to unify results.   - initial speed for the Leger test (8.5 km/h) and increases in increments of 0.5 km/h for each auditory *beep* cue;   - this rate of change between the auditory cues was used to estimate the number of shuttles ‘beeps’ completed by a child for the other three protocols.   - This information was then converted to stages and, using the Leger equation (27), stages were used to estimate maximal oxygen consumption (VO2max); the greater the number of shuttles completed indicates better performance.   - For analyses including the 20mSRT, participants from Italy and Hungary were excluded; Italian centres did not administer this test and shuttles were counted differently in Hungary and could not be reconciled using our analysis approach. |
